# Investigating Early Childhood Exclusionary Practices Within an Infant and Early Childhood Mental Health Consultation Project in the United States of America

**DOI:** 10.3390/pediatric18040098

**Published:** 2026-07-13

**Authors:** Natalia Fraczek, John S. Carlson, Jordan L. Bernard, Gillian Ogilvie, Mary Mackrain

**Affiliations:** 1 School Psychology, Michigan State University, East Lansing, MI 48824, USA; fraczekn@msu.edu (N.F.);; 2 Michigan Department of Health and Human Services, Lansing, MI 48909, USA

**Keywords:** suspension, expulsion, early childhood, exclusionary practices, infant and early childhood mental health consultation, adverse childhood experiences

## Abstract

Background/Objectives: Children in early childhood experience higher rates of suspension and expulsion than K–12 students, with persistent racial disparities. Methods: This retrospective descriptive observational study examined exclusionary practices among children ages 0–5 reported by providers participating in a state-level Infant and Early Childhood Mental Health Consultation (IECMHC) initiative within the United States. Results: Providers (*n* = 689) reported that 3.50% of children were excluded in the 12 months prior to service initiation (1.90% suspended; 1.60% expelled), with higher rates among older children (ages 3–5), males, and Black, Indigenous, People of Color (BIPOC) children. Among a subset of providers of children (n = 395) receiving child-and-family-focused (CFF) consultation, only 28 were expelled (7.09%), with the highest rate observed in children ages 30–36 months. Children expelled during CFF consultation more frequently exhibited atypical protective factors, elevated behavioral concerns, aggression at referral, and higher cumulative adverse childhood experiences (ACEs). Conclusions: Findings suggest that CFF consultation may help mitigate childcare exclusionary practices when children present with severe social-emotional-behavioral challenges. Important considerations for future childcare research and prevention efforts are provided.

## 1. Introduction

The first five years of a child’s life are the most sensitive and vulnerable time for the development of social-emotional, behavioral, and pre-academic skills [1]. When these skills fail to develop, difficult behaviors may emerge. If left unaddressed, these social, emotional, and behavioral challenges may lead to detrimental early childhood exclusionary practices [2]. Exclusion occurs when children are removed from the educational setting (e.g., suspension or expulsion). Suspension is when the child temporarily leaves the childcare setting, and expulsion is when the child permanently leaves the childcare setting. However, exclusion is more commonly used in the United States of America (USA), as in other parts of the world childcare is not compulsory. While exclusion is more commonly used in early childhood contexts, the terms suspension and expulsion, which are more often associated with schools, are used in this paper for the purposes of clarity and consistency. Research suggests that a child’s removal from early childhood settings can compromise their access to learning opportunities, making them more likely to encounter academic failure and grade retention, a negative attitude toward school, early dropout, and/or greater risk for juvenile justice system involvement [3]. Disproportionate school discipline not only pushes Black, Indigenous, and People of Color (BIPOC) students faster toward the school-to-prison pipeline but may also help to explain disparities in academic achievement when compared to White students [4,5]. These harsh disciplinary practices negatively impact children’s learning in educational contexts and functioning at home and increase stress for caregivers [6,7].

### 1.1. Rates of Suspension

Tens of thousands of preschool children are suspended from their early childcare and education settings within the USA each year [8]. Suspension prevalence rates across the USA have varied across time [9], ranging from 0.18% [10] to 6% [11]. The 2016 National Survey of Children’s Health indicated that an estimated 174,309 (2%) preschoolers ages 3 to 5 years old from across the USA were suspended [3]. Studies have also looked at state-level suspension data [12]. For example, childcare program providers in Massachusetts revealed 1.28% of preschool children aged three to four years old in childcare and public school settings had been suspended, which was less than one-fourth the rate (5.46%) for Kindergarten (K) to Grade 12 (12) in Massachusetts and less than one-fifth the rate (6.70%) for K-12 children nationally [13]. A statewide survey [14] of providers and program administrators from licensed early childcare centers and home-based programs in Colorado indicated 1.74% of young children from 0 to 6 years old had been suspended, which is slightly higher than in studies only including the preschool population. In sum, national and state-level prevalence rate studies indicate that about 2% of young children have been suspended. A closer look at national and state rates of early childhood expulsion further highlights the magnitude of exclusionary practices being used across the USA.

### 1.2. Rates of Expulsion

The National Prekindergarten Study (NPS) [15] was the first to call attention to concerning preschool expulsion rates within prekindergarten settings across the USA. Findings indicated that preschool expulsion rates (0.67%) were three times greater than expulsion rates in K-12 students (0.21%). The 2016 National Survey of Children’s Health found that more than 17,000 (0.20%) three- to five-year-olds from across the USA were estimated to be expelled annually [3]. Statewide expulsion rates ranging from 0.37% in Colorado to 2.70% in Massachusetts raise additional concerns about the frequency of young children being excluded from their early learning environments [13,14]. While several of these studies focus only on preschool ages [13], they often exclude birth to toddlerhood (up to two years old). Studies examining expulsion among children from birth to five years old in childcare settings found higher rates (35.7%) [16,17], which raises concerns about exclusionary practices during the very early childhood years. In sum, national and state prevalence studies indicate that thousands of young children are expelled annually within the USA with considerable variation by state, by child population, and by type of childcare setting. Rate variance has been linked to providers’ underreporting of expulsions since they are contrary to state policies, which are commonly focused on early childhood inclusionary practices [14]. Conversely, expulsion rates have been investigated and then utilized to advocate for state-level resources and increased support for early childhood education and early intervention services [18]. Lower expulsion rates across the USA have been linked to the availability of state-funded services, such as mental health consultation practices. Historically, mental health consultation models have been known by various names (e.g., Social Emotional Consultation, Mental Health Consultation, Early Childhood Mental Health) [19,20], but in this manuscript, the term Infant and Early Childhood Mental Health Consultation (IECMHC) will be used consistently. A closer examination of exclusionary rates within early childhood mental health support studies helps to further highlight the frequency with which these practices are utilized in behaviorally at-risk early childhood populations.

### 1.3. Prevalence of Early Childhood Exclusionary Practices Within IECMHC Studies

IECMHC is a nationally recognized evidence-based approach to meeting the social, emotional, and behavioral needs of children ages zero to five at risk for exclusion [19,21]. Within the current IECMHC model, two types of consultation services are provided. Programmatic consultation supports an entire site by equipping providers to implement evidence-based strategies across multiple children. On the other hand, Child-and-Family-Focused (CFF) consultation focuses on supporting a specific child through consultation with the provider and caregiver(s).

Suspension and expulsion rates from early childcare settings receiving IECMHC services vary considerably [17]. For example, suspension rates within a small sample (n = 47) receiving IECMHC services within a childcare setting for children aged three to six in Massachusetts was reported at 14.9% [20]. The expulsion rate within that same study was 8.5% [20], which parallels other IECMHC studies such as the 10.2% expulsion rate (2.9% expelled plus 7.3% “soft expulsion” (i.e., parent withdrew possible due to pressure from care center staff)) reported within a sample of children from birth through age five [21]. Further examination is needed to understand these rates from birth through preschool, and to explore the characteristics of those expelled during their most sensitive and formative years [20].

### 1.4. A Bioecological Framework for Understanding Exclusionary Practices

Applying a bioecological model [22] to exclusionary practices asserts that the reasons for a child’s removal from their setting are multifaceted. For example, a child’s innate biological and temperamental characteristics, their social and emotional behavior, and how those characteristics were managed by caregivers across home, school, and community contexts would all be essential to explore within the creation of early childhood expulsion prevention programs. This complex risk model highlights the importance of closely examining interactions between child (e.g., age, sex at birth, race), developmental (e.g., behavioral protective and risk factors, reason for referral, adverse childhood experiences (ACEs)), and broader ecological-level factors (e.g., childcare provider-child demographic mismatch, provider perceptions of a child’s developmental and behavioral risk) when exploring which young children are being excluded and under what conditions [23,24].

### 1.5. Child Characteristics: Age/Sex at Birth/Race

A number of child demographics have been linked to early childhood exclusionary risk [3,8]. Although no studies have found significant findings between age and suspension, older preschool children (4- and 5-year-olds) are more likely to be suspended [25]. Boys make up 54% of the students registered in public prekindergarten but are 79% of those suspended [11]. These findings indicated that boys are 3.2 times more likely to be suspended when compared to girls. African American boys comprised 18% of the preschool enrollment but are 48% of those suspended [26]. In early childhood population studies, similar findings emerged where African American boys were only 19% of the population, but 45% of those suspended [2]. Although most findings focus on the preschool population, findings indicate that BIPOC boys are about 3.6 times more likely to be suspended compared to White children in both preschool and early childcare settings [2,27].

Several demographic characteristics have also been linked to preschool expulsion. Four-year-old children are expelled at a rate of about 1.5 times greater than 3-year-olds [13]. Young boys are expelled at a rate of about 4.5 times greater than girls [27]. African American preschoolers are two times more likely to be expelled compared to Latino and White children [13]. In the birth through age five population, similar findings indicated that African American boys comprised only 8.6% of the population, but they represented 24% of those expelled [2]. The disproportionality of specific subpopulations (i.e., preschool children, males, BIPOC identifying) who are excluded from their childhood learning settings is helpful for early intervention planning purposes, but further examination is needed with younger age groups and other bioecological factors associated with this concerning societal practice [28].

### 1.6. Behavioral Protective and Risk Factors

High levels of protective factors are associated with lower behavioral problems and a lower likelihood of suspension and expulsion [29]. Protective factors (e.g., attachment behaviors and social initiation) help promote the development of positive relationships and social skills, which support children’s engagement in the home, school, and community. They are associated with healthy outcomes and building resiliency and can act as buffers when combined with challenging behaviors (e.g., emotion dysregulation), which are often associated with exclusionary practices [30]. Multi-Tiered System of Supports (MTSS) prevention models, such as the Pyramid Model, emphasize the importance of supporting social-emotional development and creating environments that foster positive behaviors. These models highlight the critical role of protective factors to reduce the need for exclusionary discipline practices [31].

Young children demonstrating high behavioral risk (e.g., impulsivity, aggression) have also been found to be at increased risk of being excluded from their learning environments [21]. Children demonstrating pervasive behavioral concerns (e.g., aggression, disruptive classroom behavior) within settings receiving IECMHC are found to be less responsive to services, leading to increased risk for suspension or expulsion despite involvement in consultative or early intervention services [32]. Further study of the connection between the targeted reason for referral (i.e., targeted behavior) and a child’s age, sex at birth, and race is warranted [23].

### 1.7. Adverse Childhood Experiences

Ecological factors such as adverse childhood events (ACEs) (e.g., abuse, neglect, family conflict, exposure to violence, household dysfunction) are known to increase the likelihood of behavioral difficulties and put a child at risk for early childhood exclusion [33]. ACEs can impact a child’s emotional regulation and overall functioning, sometimes leading to the development of post-traumatic stress symptoms or other psychological/emotional challenges. These challenges may, in turn, manifest as intense behaviors, such as aggression or emotional dysregulation, and contribute to disciplinary measures [3]. For example, a child’s chances of being suspended or expelled increased by 80% for every ACE reported. An additional study found that young children with cumulative ACE scores of four or more are four times more likely to be excluded [34,35]. In sum, children exposed to multiple adverse events may have a higher need for prevention-based services, such as IECMHC [1,3], to decrease the likelihood of suspension and expulsion.

### 1.8. Provider-Student Demographic Mismatches

In recent years, a large body of research has emerged on the mismatch between the characteristics of the providers and the children they serve in early childcare and preschool settings, suggesting that race-related perceptions of children are a strong determinant of disciplinary actions [2,36]. Identifying as a different race, such as when a child’s race differs from that of the provider, is a risk factor for exclusionary practices. This occurs when providers’ perceptions of behaviors are influenced by racial stereotypes [21]. For example, White providers are more likely than African American providers to escalate their disciplinary responses for African American students for perceived challenging behavior, even when their behaviors are comparable to behaviors exhibited by White peers [27,37].

BIPOC children’s behaviors can be perceived differently by White providers, which raises questions about differential beliefs about the nature of disruptive behaviors exhibited during the early childhood years, which are typical and can occur in about 10% to 30% of all children [38]. While providers’ perceptions can significantly impact how challenging behavior is interpreted and navigated, unaddressed disruptive behavior may lead to escalations in behavioral difficulties, which in turn can lead to harsher handling of those situations (i.e., suspensions and/or expulsions) [39]. Some challenging behaviors may have roots in influences within the child’s environment, such as exposure to traumatic events or stressful interactions with adult caregivers. These stress reactions may often appear as externalizing behavior problems such as aggression and emotional reactivity [3]. However, for some young children who are expelled, providers may struggle to identify the necessary antecedents or may utilize ineffective strategies associated with a child’s misbehavior, resulting in the use of exclusionary discipline as a means to manage classroom behavior [40]. Misperceptions around effective strategies to promote classroom safety may lead to discriminatory exclusionary discipline, especially in those situations where providers are overworked, feel overwhelmed by their responsibilities, and/or when unconscious racial biases may unintentionally impact decision-making processes.

Effective frameworks or models of consultation emphasize positive behavior support and are essential to help providers better understand and manage challenging behaviors and improve their ability to use developmentally appropriate and non-punitive strategies in response to disruptive behaviors. IECMHC is aimed to facilitate positive outcomes, reduce internalizing and externalizing behaviors in children, improve providers’ classroom management skills, and to help providers be more attuned to the meaning of children’s disruptive behaviors [19,41,42]. While IECMHC effectively promotes positive outcomes for many children [19,43], some children exit services due to expulsion, indicating the need to better understand the characteristics and needs of this subgroup and to explore whether more intensive or tailored support may be beneficial [36,44].

### 1.9. Present Study

This study explored suspension and expulsion prevalence data reported by providers seeking consultation support within a state infant and early childhood mental health consultation (IECMHC) project. The full analytic sample consisted of provider-reported data on children served across participating childcare settings. Within this overall sample, two distinct subgroups were examined. First, demographics (age, sex at birth, race/ethnicity) of children reported by providers to have experienced suspension (temporarily left the childcare setting) or expulsion (permanently left the childcare setting due to expulsion) in the 12 months prior to initiating IECMHC services (CFF or programmatic consultation) were analyzed to describe pre-service exclusionary experiences. Second, a separate subsample of children who received individualized CFF consultation was identified to examine bioecological factors (age, sex at birth, race/ethnicity, presence of protective and risk factors, reason for referral, ACEs, provider race/ethnicity) of children that exited services due to expulsion during consultation. Importantly, these two groups are not mutually overlapping analytic comparisons, but rather represent distinct timepoints and service contexts within the broader IECMHC dataset. Based on prior literature on exclusionary discipline in early childhood settings, it was hypothesized that boys, older preschool-aged children, and children identified as BIPOC would be disproportionately represented among children reported to have experienced suspension or expulsion prior to IECMHC services. It was also hypothesized that children with elevated behavioral concerns, lower protective factors, higher ACE exposure, and referrals related to challenging behaviors would be more likely to exit CFF consultation due to expulsion.

Demographics (age, sex at birth, race/ethnicity) of children reported by providers to have been suspended (temporarily left the childcare setting) or expelled (permanently left the childcare setting due to expulsion) in the 12 months prior to providers seeking CFF or programmatic consultation were examined. Analyses were conducted to understand the characteristics of children who were suspended and expelled from their placement prior to the implementation of IECMHC services. Separately, an additional subgroup of children and their providers who engaged in CFF consultation were identified. An array of bioecological factors (age, sex at birth, race/ethnicity, presence of protective and risk factors, reason for referral, ACEs, provider race/ethnicity) that may be associated of expulsion were explored in this subsample of CFF Consultation cases. In sum, these two groups provide an examination between children excluded before receiving IECMHC services (e.g., CFF or programmatic consultation) and those who exited services due to expulsion while actively engaged in individualized CFF consultation. Specifically, this study addressed the following research questions:

1. What percentage of children were reported to be excluded (i.e., suspension or expulsion) from their childcare settings in the past 12 months before receiving IECMHC services (CFF or programmatic consultation)?
  (a) What was the child-level demographic (i.e., age, sex at birth, race/ethnicity) breakdown of those who were excluded (i.e., suspension or expulsion)?
  (b) Which child-level demographic variables (i.e., age, sex at birth, race/ethnicity) best associate those who were reported to be excluded (i.e., suspension or expulsion)?
2. Of children who began CFF consultation (i.e., parent consent for services was obtained), what percentage was reported by providers to have exited due to expulsion?
  (a) What was the breakdown of the bioecological variables (i.e., age, sex at birth, race/ethnicity, presence of protective and risk factors, reason for referral, ACEs, provider race/ethnicity) of those who exited CFF consultation due to expulsion?
  (b) Which bioecological variables (i.e., age, sex at birth, race/ethnicity, presence of protective and risk factors, reason for referral, ACEs, provider race/ethnicity) best associate those who exited CFF consultation due to expulsion?

## 2. Method

### 2.1. Participants

This retrospective descriptive observational study used exclusionary discipline data gathered from childcare providers who sought IECMHC services (CFF or programmatic Consultation; January 2019 to September 2023) within an early intervention initiative were utilized to address the first research question. Across 19 agencies, a total of 689 childcare providers participated in IECMHC services. Across both types of IECMHC services (CFF or programmatic consultation), providers predominantly identified their racial identity as White (84%), while the remaining 16% identified as BIPOC (Table 1). The providers in this sample came from a variety of early childhood education settings, including center-based programs (84%), home-based programs (15%), Great Start Readiness programs (0.90%), and family-friend programs (0.15%). These programs use a rating system aimed at setting high quality early learning standards and evaluating the quality of early care programs. Programs had a diverse array of ratings (zero = poor; three = average; five = excellent). Nineteen percent had less than two stars, 52% had three stars, and 29% were rated as having four or five stars.

A sample of 8856 young children for whom providers then reported the frequency of exclusionary practices (e.g., suspension or expulsion) in their classrooms over the 12 months leading up to consultation was reported. Provider report of the children’s ages was captured within four age categories: birth–30 months old (26%), 30 months–3 years old (16%), 3–4 years old (29%), and 4–5 years old (28%). While sex at birth and race data were not specifically reported by providers for children served (n = 8856), sample estimates of those demographics were calculated based on state population statistics for birth-5-year-olds [45]. The percentages shown in Table 2 reflect the population statistics and sample size estimates. About half of the children were reported to be male (51%) compared to female (49%), and the majority were White (67%) compared to BIPOC (33%).

To address research question two, 397 providers (58%) reported 395 children receiving CFF consultation (Table 3). Data on those who exited services due to expulsion from the childcare placement while receiving programmatic consultation were not available. Only those who exited services due to expulsion from placement while receiving CFF consultation was available, and therefore, was examined for this research question. While CFF consultation is typically delivered by one mental health consultant to the childcare providers working with a child identified to need individualized consultation, in two cases, each child received support from two providers. The children were a range of ages from birth–30 months old (16%), 30 months–3 years old (11%), 3–4 years old (35%), and 4–5 years old (37%) with the majority being male (77%). The racial identity of the majority of children were White (76%), with smaller percentages identifying as BIPOC (24%). Race and ethnicity data were originally collected using the following categories: White, African American/Black, Hispanic/Latino, multiracial/biracial, and more than one race. Due to small subgroup sample sizes that limited statistical analyses, children identified as African American/Black, Hispanic/Latino, multiracial/biracial, or more than one race were collapsed into a single BIPOC category for analyses, while children identified as White were retained as a separate category. Collapsing multiple racial and ethnic groups into a single BIPOC category may obscure important differences in experiences and outcomes across groups and limits the ability to examine culturally specific patterns of exclusion. On average, the children who were expelled received about seven months of CFF consultation (mean = 6.78 months, standard deviation = 5.88, range= 0.73–21.40 months). The analysis for this research question solely focused on expulsion, as suspension data were not captured.

### 2.2. Measures

Data Collection Form. To collect information on children referred to IECMHC services (i.e., CFF or programmatic consultation) consultants completed a demographic form before service initiation. This form gathered data on the number of children suspended or who exited services due to expulsion from the childcare placement in the 12 months prior to initiation of CFF or programmatic consultation, along with their demographic characteristics reported by the childcare provider. This data also included the child’s age, sex at birth, race, and ethnicity during the pre-assessment, as well as the race and ethnicity of the providers. Additionally, for the subset of children who received direct CFF consultation, information was collected on why services were discontinued. Consultants were able to select from a range of options that included achievement of consultation goals, the childcare site closed, the child left the provider, the child was expelled, the provider no longer wished to receive consultation, the provider transitioned to another consultant, the consultant left and the provider did not want to transition to a new consultant, the child was transitioned to special education or a more appropriate setting, or the consultant was unable to contact the provider. However, for the purposes of this study, only cases in which services were discontinued due to expulsion were analyzed to explore the characteristics of that subsample of children.

Due to sample size and missing data, responses regarding race and ethnicity were categorized into the following groups: White and BIPOC. For the purpose of this study, ages were grouped as follows: birth to 30 months, 30 months to 3 years, 3 to 4 years, and 4 to 5 years. Sex at birth was classified as a binary variable: male and female.

The Devereux Early Childhood Assessment Program for Infants and Toddlers (DECA-I [46]; DECA-T [47]). The Devereux Early Childhood Assessment for Infants and Toddlers (DECA-I and DECA-T) is a caregiver and provider-report measure designed to assess infants’ and toddlers’ social-emotional protective factors, including attachment/relationship quality, initiative, and self-regulation [46,48]. The DECA-I (for ages 4 weeks up to 18 months) consists of 33 questions that contribute to a Total Protective Factors scale and two subscales (Attachment/Relationship and Initiative). The DECA-T (for ages 18 to 36 months) consists of 36 questions that contribute to a Total Protective Factors scale and three subscales (Attachment/Relationship, Initiative, and Self-Regulation). In the present study, the DECA-I and DECA-T were completed by caregivers (e.g., biological or foster parents) or childcare providers as part of the CFF consultation process to assess child social-emotional functioning and protective factors. Both have shown high internal (0.90–0.95), test–retest (0.72–0.98), and inter-rater (0.62–0.74) reliability. Construct, criterion, and content validities have also been demonstrated [47].

The Devereux Early Childhood Assessment Clinical Form (DECA-C: [49]). The DECA-C [49] is a caregiver and provider-report measure designed to assess young children’s social-emotional strengths and behavioral concerns, including initiative, self-control, attachment, aggression, withdrawal/depression, and attention problems. The DECA-C [49] is intended for children ages 2 through 5 years and consists of 62 items across two domains: Protective Factors and Behavioral Concerns. The Protective Factors consists of 27 items (three subscales: Initiative, Self-Control, and Attachment), and the Behavior Concerns scale consists of 35 items (four subscales: Aggression, Increased Concern, Withdrawal/Depression, and Attention Problems). Protective Factors scores are categorized as either typical or atypical, with atypical scores reflecting lower-than-expected social-emotional strengths relative to same-age peers. Behavioral Concerns scores are also categorized as either typical or atypical, with atypical scores reflecting elevated levels of behavioral and emotional concerns relative to same-age peers. In the present study, Protective Factors T-scores from the DECA-I, DECA-T, and DECA-C caregiver and provider forms were combined into a single variable to create one unified score for analysis across the full early childhood age range. Scores are reported as T-scores ranging from 28 to 72, with a mean of 50. The DECA-C has demonstrated high internal (0.88–0.94), test–retest (0.74–0.94), and inter-rater (0.66–0.69) reliability. Construct, criterion, and content validities have also been established [49].

The Center for Youth Wellness Adverse Childhood Experiences (ACEs) Questionnaire (CYW ACE-Q [50]). The ACE-Q [50] section one includes ten questions that assess childhood trauma, including those that are experiences of the child and experiences or behaviors of close family members. There is an additional section two with seven ACE questions that assess exposure to early childhood life stressors applicable to children served in community settings [51], which can be scored separately or combined. Combined sections help to determine who is at low, intermediate, or high risk for toxic stress [51]. Section one demonstrates good test–retest reliability (0.79), internal consistency (0.72), and concurrent validity [52], but section two is not currently a validated diagnostic tool [51,53]. Although section two has not been validated as a diagnostic measure, it was included in the present study to capture contextual stressors relevant to the experiences of children receiving CFF services. Consistent with prior use of the CYW ACE-Q, sections one and two were used to identify cumulative exposure to adversity and toxic stress risk rather than for diagnostic classification.

### 2.3. Procedure

IECMHC consultants worked closely with providers to complete the Data Collection Form. This form was used to gather demographic information about providers and children. For inclusion in the present study, cases were required to have complete records indicating (a) confirmation of IECMHC services (CFF or programmatic consultation) and (b) provider-reported information on child exclusionary outcomes (suspension or expulsion) within the relevant time period. No additional exclusion criteria were applied beyond missing outcome data, which resulted in case-level variation in available demographic information across analyses.

To address the first research question, this set of data was used to summarize the demographics of children who faced suspension or expulsion 12 months before services (i.e., IECMHC CFF or programmatic consultation) began. For the children who received CFF consultation, the Data Collection Form also gathered demographic information about both providers and children. To address the second research question, a distinct subsample was created consisting only of children who received individualized CFF consultation and for whom service termination data were available. Within this subsample, information was summarized to better understand the bioecological variables associated with children who had exited services due to expulsion from their early childhood setting while engaged in CFF consultation.

The data used to evaluate the research questions was extracted from an extant dataset and is not linked to any identifiable information. This de-identified dataset was extracted from a web-based database via an exported Excel file, which was then imported to IBM SPSS Statistics (Version 28) [54] for data analysis. This study was determined to meet the criteria for non-human subject research by the Office of Regulatory Affairs Human Research Protection Program board on 9 November 2023 (STUDY00009914).

## 3. Data Analysis

Data analyses were conducted in two stages corresponding to the two research questions. First, for the full provider-reported sample, the proportion of children reported to have experienced suspension or expulsion in the 12 months prior to receiving IECMHC services (CFF or programmatic consultation) was calculated using the total number of children with available outcome data as the denominator. Child-level demographic characteristics (age, sex at birth, race/ethnicity) for this subgroup were then summarized using frequency distributions to describe the composition of children who experienced exclusionary outcomes.

Second, for the CFF consultation subsample, the proportion of children who exited services due to expulsion was calculated using all children who received CFF consultation as the denominator. Within this subsample, bioecological variables (age, sex at birth, race/ethnicity, protective and risk factors, reason for referral, ACEs, and provider race/ethnicity) were summarized using descriptive frequencies. A one-sample chi-square goodness-of-fit test was conducted to examine whether observed distributions differed from expected population distributions.

### 3.1. Child Demographics of Children Excluded (i.e., Suspended/Expelled) in the Past 12 Months

To address research question one, all cases were included in the overall prevalence calculations (e.g., suspensions and expulsions); however, missing demographic data resulted in slightly smaller sample sizes for analyses related to age, sex at birth, and race/ethnicity. Specifically, demographic data on 16 children excluded for sex at birth and 30 for race/ethnicity were not indicated by the responding providers. These data were not provided by responding providers at the time of data collection and appear to be sporadic and not associated with any specific site, program, or child characteristics available in the dataset. No imputation procedures were used; analyses were conducted using available cases for each variable (listwise deletion by variable). Descriptive frequency analyses were used to determine the breakdown of those reported to have been suspended or expelled in the 12 months prior to providers seeking IECMHC services. Since no direct comparison group was available for children who experienced exclusion in the 12 months prior to consultation, proxy comparison data were used. For age, a comparison distribution was derived from the full IECMHC sample (CFF and programmatic consultation), which included age information for all children. However, sex at birth and race/ethnicity were not available in this dataset. Therefore, for these variables, expected distributions were estimated using population-level proportions from the 2021 Michigan Kids Count Data Center [45] for children ages 0–5. To calculate these estimates, demographic proportions (e.g., percentage of males/females or percentage of each racial/ethnic group) were used from the state-reported data. These proportions were then applied to the total number of children receiving CFF consultation. For example, to estimate the number of males in the CFF group, the percentage of males in the state population was multiplied by the total number of children receiving CFF consultation. This approach was similarly applied to race/ethnicity, using the corresponding percentages for each group from the state data. These estimates allowed for comparison of observed versus expected distributions; however, findings should be interpreted cautiously, as state-level population data do not represent a matched or service-equivalent comparison group and therefore do not support causal inference. Expected counts were therefore derived from either the IECMHC sample (for age) or state-level population data (for sex at birth and race/ethnicity). Analyses were conducted separately by type of exclusion (suspension or expulsion) and by child-level demographic characteristics (age, sex at birth, and race/ethnicity) using one-sample chi-square goodness-of-fit tests to compare observed and expected distributions.

### 3.2. Bioecological Variables of Children Expelled While Engaged in CFF Consultation

To address research question two, all cases were included in the overall expulsion prevalence calculations and for analyses related to bioecological characteristics, including age, sex at birth, race/ethnicity, presence of protective and risk factors, reason for referral, ACEs, and provider race/ethnicity. Descriptive analyses were conducted on the group who was engaged in CFF, specifically to ascertain the proportion of children identified by providers who have exited services due to expulsion from their childcare setting. Bioecological variables were analyzed, and the data was disaggregated to explore potential disparities among children who exited services due to expulsion from their setting while receiving consultation services. Observed frequencies and expected counts were gathered based on population numbers. A one-sample chi-square goodness-of-fit test was conducted to examine any disproportionate representations of bioecological characteristics within the expelled group during consultation services.

## 4. Results

**Figure 1:**
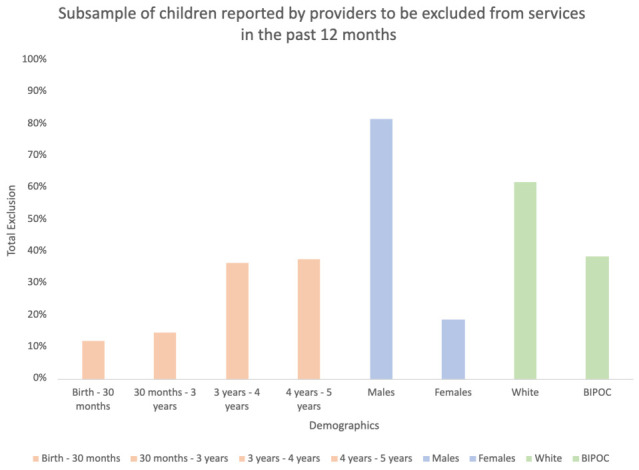
Subsample of children reported by providers to be excluded from services in the past 12 months.

A total of 689 providers across 19 agencies reported 309 children (3.50%) had been the subject of an exclusionary practice of the 8856 children served in the 12 months prior to the onset of IECMHC services. Table 1 summarizes the providers and settings involved. To explore statistically significant demographic associations associated with exclusion, a one-sample chi-square goodness-of-fit test found a significant overrepresentation of older children (χ^2^ = 36.79, df = 3, *p* < 0.01), males (χ^2^ = 131.93, df = 1, *p* < 0.01), and BIPOC children (χ^2^ = 30.61, df = 1, *p* < 0.01). Children aged 4–5 years had the highest exclusion rate at 4.44% when compared to the overall rate of 3.50% for the entire sample. The rate for 3–4-year-olds at 4.07% was also higher than expected, while younger children were less frequently excluded compared to the other age groups (30 months to 3 years = 2.94%; birth to 30 months = 1.50%). Males too were overrepresented among those excluded when compared to females, at a rate of 5.28% vs. 1.24%. BIPOC children too were excluded more frequently at a rate of 3.66% compared to 2.90% for White (Figure 1). Since suspension and expulsion data were aggregated for this analysis, statistical significance testing for each exclusion type was not performed. Disaggregation of data by exclusionary type in Table 2 highlights that similar trends were observed for suspensions, with the highest rates reported among the oldest age group of children (aged four to five years old), males, and those identified as BIPOC. It is important to note that missing demographic data account for the reduced Ns for excluded subgroups reflected in Table 2.

In addition to the rate and characteristics of those excluded prior to receiving IECMHC, a smaller subsample of children who exited services due to expulsion from their childcare setting while engaged in CFF consultation was examined. A total of 23 providers reported 28 instances (7%) of expulsion from among the 395 children who received CFF. Table 3 summarizes the bioecological factors (e.g., age, sex at birth, race/ethnicity, reason for referral, protective factors, behavioral concerns, ACEs, provider race/ethnicity) of those expelled compared to the entire sample of those receiving CFF consultation. An analysis revealed no statistically significant disparities in prevalence rates. However, trends appeared to indicate younger children (30 months to 3-year-olds) were expelled at a disproportional rate (11.36%) compared to the other age group rates: birth to 30 months (8.06%), 4–5-year-olds (7.04%) and 3–4-year-olds (5.93%). Males had a higher expulsion rate (7.88%) compared to females (5.31%). BIPOC children were expelled at a disproportionately higher rate (10.23%) compared to White children (6.74%). Additionally, higher exclusion rates were associated with caregiver and provider ratings on the three DECA measures [46,47,49], indicating atypicality (parent form = 5.56% indicated atypical protective factors; provider form = 8.21% indicated atypical protective factors) compared to ratings indicating typical protective factors (parent form = 4.44% indicated typical protective factors; provider form = 4.31% indicated typical protective factors). Expulsion was also higher in the caregiver group who rated their children as having atypical behavioral concerns (parent = 7.31% indicated atypical behavioral concerns; provider = 6.66% indicated atypical behavioral concerns) compared to typical behavioral concerns (2.86% v. 4.31% indicated typical behavioral concerns). There was a considerably higher rate of expulsion in aggression/physical-related referrals (10.14%) compared to other referral types, a noticeably higher rate for those children who experienced three or more ACEs (7.35%) compared to less than three ACEs on sections one and two (0.94%), and a slightly higher rate among children cared for by BIPOC (9.53%) providers compared to White providers (6.71%).

## 5. Discussion

A sample of providers seeking IECMHC services reported a total exclusion (i.e., suspension or expulsion) rate of 3.50% in the children being served in their classrooms in the twelve months prior to seeking IECMHC services (i.e., CFF or programmatic consultation). It is important to note that the provider-reported data on exclusionary practices did not allow for identifying children who may have experienced both suspension and expulsion. State and national data on early childhood exclusion typically separate suspension and expulsion rates. Given that this is the first published study to report on a combined exclusionary rate within the early childhood population, it is difficult to contextualize within the literature. However, this study also examined each exclusionary practice (e.g., suspension, expulsion) independently for a more detailed analysis to understand potential differences in the characteristics of affected children and the contexts in which each occurred. Furthermore, these rates must be considered within the context of the at-risk population, who may experience increased behavioral challenges or other underlying stressors, common with referral to and involvement with IECMHC. Additionally, the COVID-19 pandemic, which was ongoing during some of this period, likely exacerbated these stressors and could have further influenced behaviors or circumstances in ways that are important to consider contextually [55].

Suspension rates (1.90%) reported by 689 providers seeking IECMHC services (i.e., CFF or programmatic consultation) were generally consistent with prior studies involving state-based samples of young children (1.28% to 1.74%) [13,14]. The slightly higher rate of suspension, when compared with other state-based samples, may be the result of the level of risk associated with this population of providers who were seeking consultation services to assist with an identified challenge within their setting. Additionally, Gilliam and Shahar’s study [13] focused exclusively on a preschool sample, which may account for their slightly lower suspension rates. In contrast, Miles and colleagues study [14] examined children from birth to age six in early childhood settings, which may explain why their suspension rate was more comparable to ours, suggesting that the 1.90% rate observed in our study may reflect a typical rate for this early childhood age range. When looking at rates from national samples (0.18% to 6%) [10], our suspension rate fell within the lower range of reported estimates. This may reflect differences in sample composition, as national estimates include a wide variety of early childhood settings with varying structures, resources, and levels of child and family risk [18].

Expulsion rates (1.60%) reported by providers who were seeking IECMHC services (i.e., CFF or programmatic consultation) were in the lower range of rates reported in other states (0.37% [13]; 2.70% [14]). Compared to national samples across the United States (0.21% [15]; 0.20% [3]), our rate was higher; however, these national studies included various settings and typically only the preschool population, not infants and toddlers. Given that our sample included a wider variety of ages and is a more at-risk subgroup where providers were seeking IECMHC support, this study sample may reflect increased needs or higher levels of support required compared to the broader preschool population. The literature suggests that while K-12 schools primarily resort to suspension, early childhood settings might lean towards a quicker and more permanent solution provided by expulsion, especially if children exhibit behavioral challenges [13]. Delays in accessing support services may also contribute to the expulsion rate, suggesting that while IECMHC can potentially prevent future expulsions, many of these children reported in this study may have faced exclusion prior to their providers receiving the consultative support they may have needed.

Several groups of children were identified as being disproportionally excluded (i.e., suspension, expulsion) by our sample of providers seeking IECMHC services, with similar trends seen for both suspensions and expulsions within this same group. For example, older children experienced higher rates of exclusion compared to other demographic subgroups. This aligns with prior research findings showing that the risk for exclusion increases as children approach the later early childhood years [15]. The developmental stage of preschoolers (3–5 years old) may contribute to their perceived defiance as they navigate increased demands and expectations. Male children also experienced higher rates of exclusion compared to female children. This is consistent with the prior literature demonstrating that boys are more likely to experience suspension and expulsion in early childhood settings [27]. Additionally, BIPOC boys face a heightened risk for exclusion [2,26,27] and have been known to receive suspensions and expulsions at disproportionate rates [5,56]. This finding adds nuance to the existing literature by demonstrating similar disproportionalities in suspension and expulsion rates [13,14] within a unique at-risk group of children whose providers were entering IECMHC services.

Among children who received CFF consultation due to serious social-emotional-behavioral concerns, providers reported only 7% were expelled from their childcare setting. This rate is slightly lower than three rates reported in previous studies within childcare settings, including one indicating an 8.6% rate [41], another 8.5% [20], and a more recent one indicating up to 10.3% when considering expulsions and parent withdrawals (i.e., “soft expulsions”) from the childcare setting. However, our expulsion rate should be interpreted cautiously given the descriptive nature of the present study and the small CFF subsample (n = 28). This rate is also considerably lower than the extraordinary 35.7% incidence (i.e., last 12 months) rate of expulsion reported by administrators in one state’s community childcare centers [16] and the 42% rate of childcare centers who reported expelling at least one infant or toddler within the past year [57]. Such rate differences between typical community-based childcare and others that may have access to IECMHC services (i.e., CFF or programmatic consultation) highlight a possible association between access to these supports and lower use of exclusionary practices. Parallel conclusions have been made in prior studies that found a significant decrease in expulsions when teachers had access to IECMHC [17,58]. Specifically, prior research has reported improvements in children’s functioning and lower expulsion risk among children receiving CFF consultation [59]. Ultimately, CFF consultation may be associated with lower rates of expulsion by providing targeted, intensive support where it is needed most; however, these findings should be interpreted cautiously, as they are descriptive and hypothesis-generating rather than evidence of a causal relationship.

Unlike other studies that focused solely on demographic factors to identify at-risk groups for expulsion, this study considered multiple bioecological factors that may be associated with childcare expulsion while receiving CFF consultation. This broader range of influences offers a more comprehensive understanding of the complex dynamics contributing to expulsion. Although the results did not show statistical significance, the small sample size (N = 28) likely accounted for this limitation. Interestingly, while older children (ages 4–5) were more likely reported by providers to be excluded (suspended or expelled) in the 12 months prior to receiving IECMHC services, expulsion rates during CFF consultation were highest among younger children aged 30 months to 3 years old. This could be due to the developmental challenges characterized by this age group, such as increased behavioral outbursts, delays in oral expression, and/or difficulties in emotional regulation. This finding was unexpected and highlights the importance of considering age-specific factors when identifying at-risk children for expulsion. Males and BIPOC children also had the highest rates of expulsion, consistent with prior studies [13,28], which have consistently highlighted racial and gender disparities in early childhood expulsion rates.

Expulsion rate trends from this study highlight additional bioecological factors that may put young children at risk for exclusionary practices. Specifically, parent and provider ratings indicated a high prevalence of atypical protective factors compared to typical protective factors, and a high prevalence of atypical behavioral concerns compared to typical behavioral concerns. There was also a high prevalence of aggression-related referrals compared to other referral types, a higher rate for those children who experienced three or more ACEs compared to less than three ACEs, and a slightly higher rate among children cared for by providers who identified as BIPOC. The high prevalence of atypical protective factors and behavioral concerns, aggression-related referrals, and cumulative ACEs among young children may indicate challenging behavior, potentially contributing to difficulties managing behavior in the classroom, increasing the likelihood of expulsion [21,29].

### Limitations and Future Research

The current study is subject to several limitations. First, it is important to note that this study was conducted during the COVID-19 pandemic, a period marked by significant disruptions and additional stressors. These factors may have influenced behaviors and exclusionary practices and, therefore, should be considered when interpreting the findings. Second, although exit reasons were collected, including expulsion, we do not know the reason a child was expelled. It may be possible that the student was expelled for reasons beyond those highlighted above, which may not include disruptive behaviors. The absence of information regarding the reasons for exclusion limits the ability to determine whether expulsions were directly related to behavioral concerns or other contextual factors. Third, seven (25%) children who had been expelled had providers/parents who had received CFF consultation for a duration of less than two months, which may be consistent with the belief that teachers often ask for help at the same time they are seriously considering removal from the placement. Some children may have already been at elevated risk for exclusion prior to the initiation of services, limiting the ability to determine the extent to which consultation influenced expulsion outcomes. Additionally, children who had already experienced suspension or expulsion prior to the initiation of IECMHC services may no longer have been present in the childcare setting when consultation began, thereby reducing the number of children remaining at risk for subsequent exclusion during CFF consultation. Fourth, determining whether the suspension and expulsion rates are low, typical, or high is challenging because comparison studies differ in child age ranges, types of early childhood settings, and the level of risk of the populations studied. As a result, interpreting these rates within the broader literature requires caution. Fifth, sex-at-birth and race/ethnicity population data were obtained from a state database and used as proxy demographic comparison data when examining suspensions and expulsions reported by providers seeking services. As such, the use of proxy demographic data represents a study limitation. Specifically, suspension and expulsion rates were calculated using sex-at-birth and race/ethnicity population estimates derived from the state database rather than directly from the full population served. The lack of a control group for sex at birth and race/ethnicity for research question one limits the internal validity of the findings. Due to the small sample size, race and ethnicity categories were merged for this analysis. However, separating race and ethnicity is essential for future research as they represent distinct identities. Finally, when looking at those who exited services due to expulsion from their childcare setting while engaged in CFF consultation, the statistical power was limited because of the small sample size of children expelled (n = 28). Thus, the analysis needed more power to detect if the observed distribution of categorical data significantly deviates from the expected distribution. Additionally, missing demographic data reduced subgroup sample sizes for several analyses and may have limited the interpretation and generalizability of findings.

Future research should consider the influence of distal factors such as the socio-economic status, the community children live in, family dynamics, and access to resources, as suggested by existing literature [43,60]. Cultural expectations and values across different contexts (e.g., home, school, and community) are important as well because cultural norms may shape how children’s behaviors are perceived and managed. These differences could impact how exclusionary practices are applied and interpreted, potentially affecting both the outcomes of such practices and how they are perceived. These broader contextual factors may play a significant role in shaping a child’s behavior and interaction with educational environments, potentially putting them at greater risk of exclusionary practices.

## 6. Conclusions

Given the characteristics of the two different samples explored, this study contributes uniquely to prior studies focused on the complex dynamics and intricacies surrounding preschool suspension and expulsion. It offers additional insight into the at-risk early childhood population, and notes observed disparities, and a persistent vulnerability faced by both older children (3 to 5 years old), who were more likely to experience suspension or expulsion before providers sought IECMHC services, and the younger children (ages 30 to 36 months), who had the highest expulsion rates from their childcare setting while engaged in CFF consultation. Additionally, males, BIPOC children, those with lower protective factors, higher behavioral concerns, aggression at referral, and a higher number of cumulative ACEs showed notable disparities in expulsion rates that warrant further attention. This study’s unique focus on bioecological factors that are beyond basic demographic characteristics provides a comprehensive approach critical to fostering inclusive and supportive early childhood education environments and providing more targeted and effective mental health services. From an implementation science perspective, IECMHC services (i.e., CFF and programmatic consultation) appear to help mitigate exclusionary discipline rates [61]. Study findings may help to further identify important targets for prevention and early intervention. Overall, study findings contribute to the advancement of IECMHC to reduce exclusionary practices and promote equitable access to early childhood education.

## Figures and Tables

**Table 1:** Provider demographics and program characteristics associated with CFF or programmatic consultation.

		Programmatic	CFF	Total
Characteristic		*N* = 292	*N* = 397	*N* = 689
		*n* = 238	*n* = 321	*n* = 545
Provider Race/Ethnicity	White	185 (77.73%)	268 (83.49%)	459 (84.22%)
	BIPOC	53 (22.27%)	53 (16.51%)	86 (15.78%)
		*n* = 238	*n* = 311	*n* = 548
Star Rating	0	16 (6.72%)	31 (9.97%)	47 (8.58%)
	1	8 (3.36%)	29 (9.32%)	36 (6.57%)
	2	9 (3.78%)	11 (3.54%)	20 (3.65%)
	3	140 (58.82%)	144 (46.30%)	284 (51.82%)
	4	48 (20.16%)	73 (23.47%)	121 (22.08%)
	5	17 (7.14%)	23 (7.40%)	40 (7.30%)
		*n* = 279	*n* = 384	*n* = 663
Program Type	Home	54 (19.35%)	43 (11.20%)	97 (14.63%)
	Center	223 (79.93%)	336 (87.50%)	559 (84.31%)
	GSRP	1 (0.36%)	5 (1.30%)	6 (0.90%)
	Family Friend	1 (0.36%)	-	1 (0.15%)

BIPOC: Black, Indigenous, and People of Color.

**Table 2:** Demographic summary of the population and the subsample of children reported by providers to be excluded from services in the past 12 months.

		Suspended *N* (%)	Expelled *N* (%)	Total Exclusion *N* (%)	*p*-Value	Population of Children *N* (%)
Total		167 (1.90%)	142 (1.60%)	309 (3.50%)		8856
Age		*n* = 159	*n* = 134	*n* = 293	<0.01	
	Birth–30 months	17 (10.70%)	18 (13.43%)	35 (11.95%)		2340 (26.42%)
	30 months–3 years	16 (10.06%)	26 (19.40%)	42 (14.33%)		1429 (16.13%)
	3 years–4 years	56 (35.22%)	50 (37.31%)	106 (36.18%)		2607 (29.43%)
	4 years–5 years	70 (44.02%)	40 (29.85%)	110 (37.54%)		2480 (28.00%)
Sex at Birth		*n* = 167	*n* = 128	*n* = 293	<0.01	
	Males	134 (80.23%)	103 (80.47%)	239 (81.56%)		4534 ^+^ (51.2%)
	Females	33 (19.76%)	25 (19.53%)	54 (18.43%)		4322 ^+^ (48.8%)
Race/Ethnicity		*n* = 157	*n* = 130	*n* = 279	<0.01	
	White	97 (61.78%)	75 (57.69%)	172 (61.64%)		5934 ^+^ (67.00%)
	BIPOC	60 (38.21%)	55 (42.30%)	107 (38.35%)		2922 ^+^ (33.00%)

^+^ Sample demographic estimates for sex at birth and race/ethnicity are based on population-based statistics [45].

**Table 3:** Rates of those reported expelled, by bioecological demographic variable, compared to the overall group of those receiving CFF consultation.

		Expelled	*p*-Value	Total Children Receiving CFF Services	* Expulsion Rate
Total		*N* = 28		*N* = 395	7.09%
Age		*n* = 28	0.769	*n* = 383	
	Birth–30 months	5 (17.86%)		62 (16.19%)	8.06%
	30 months-3 years	5 (17.86%)		44 (11.49%)	11.36%
	3 years–4 years	8 (28.57%)		135 (35.24%)	5.93%
	4 years–5 years	10 (35.71%)		142 (37.07%)	7.04%
Sex at Birth		*n* = 28	0.083	*n* = 386	
	Males	23 (82.14%)		292 (75.64%)	7.88%
	Females	5 (17.86%)		94 (24.35%)	5.31%
Race/Ethnicity		*n* = 28	0.416	*n* = 370	
	White	19 (67.86%)		282 (76.21%)	6.74%
	BIPOC	9 (32.14%)		88 (23.78%)	10.23%
DECA Parent		*n* = 15	0.546	*n* = 306	
	Protective Factors Typical	8 (53.33%)		180 (58.82%)	4.44%
	Protective Factors Atypical	7 (46.66%)		126 (42.18%)	5.56%
		*n* = 14	0.195	*n* = 234	
	Behavioral Concerns Typical	2 (14.29%)		70 (29.91%)	2.86%
	Behavioral Concerns Atypical	12 (85.71%)		164 (70.09%)	7.31%
DECA Provider		*n* = 22	0.219	*n* = 323	
	Protective Factors Typical	5 (22.72%)		116 (35.91%)	4.31%
	Protective Factors Atypical	17 (77.27%)		207 (64.99%)	8.21%
		*n* = 16	0.783	*n* = 247	
	Behavioral Concerns Typical	2 (12.5%)		34 (13.77%)	5.88%
	Behavioral Concerns Atypical	14 (87.5%)		213 (86.23%)	6.66%
Referral Reason		*n* = 28	0.856	*n* = 371	
	Aggression	20 (71.42%)		203 (54.72%)	9.85%
	Developmental	3 (10.71%)		57 (15.37%)	5.26%
	Externalized Behaviors	0 (0.00%)		8 (2.16%)	0.00%
	Physical	1 (3.57%)		4 (1.08%)	25.00%
	Regulatory	4 (14.29%)		93 (25.07%)	4.30%
	Sensory Integration	0 (0.00%)		6 (1.62%)	0.00%
ACEs Section one		*n* = 10	0.520	*n* = 386	
	Score: 0	3 (30.00%)		199 (51.55%)	1.51%
	Score: 1	3 (30.00%)		94 (24.35%)	3.19%
	Score: 2	2 (20.00%)		41 (10.62%)	4.88%
	Score: 3	0 (0.00%)		27 (6.99%)	0.00%
	Score: 4+	2 (20.00%)		25 (6.48%)	8.00%
ACE Sections one + two		*n* = 8	0.645	*N* = 366	
	Score: 0	2 (25.00%)		183 (50.00%)	1.09%
	Score: 1	1 (12.5%)		83 (22.68%)	1.20%
	Score: 2	0 (0.00%)		52 (14.21%)	0.00%
	Score: 3	2 (25.00%)		27 (7.38%)	7.41%
	Score: 4+	3 (37.50%)		41 (11.20%)	7.32%
Provider Race/Ethnicity		*n* = 23	0.566	*n* = 321	
	White	18 (78.26%)		268 (83.49%)	6.72%
	BIPOC	** 5 (21.73%)		53 (16.51%)	9.43%

* Expulsion rates represent the proportion of children expelled within each subgroup (number expelled ÷ total number of children in that subgroup × 100). Group differences in expulsion status were examined using Fisher’s exact tests, as appropriate based on cell counts. ** Percentages based on small cell sizes (*n* < 5), including the Physical referral category (*n* = 4), should be interpreted with caution due to instability of estimates.

## Data Availability

The datasets presented in this article are not readily available due to data sharing restrictions. Requests to access the dataset should be directed to the corresponding author at carlsoj@msu.edu.
